# Regulating Water Reduction Kinetics on MoP Electrocatalysts Through Se Doping for Accelerated Alkaline Hydrogen Production

**DOI:** 10.3389/fchem.2021.737495

**Published:** 2021-10-01

**Authors:** Zhenpeng Liu, Jun Bu, Wenxiu Ma, Bin Yang, Lei Zhang, Hong Zhong, Shuangming Li, Jian Zhang

**Affiliations:** ^1^ State Key Laboratory of Solidification Processing, Northwestern Polytechnical University, Xi’an, China; ^2^ Key Laboratory of Special Functional and Smart Polymer Materials of Ministry of Industry and Information Technology and Department of Advanced Chemical Engineering, School of Chemistry and Chemical Engineering, Northwestern Polytechnical University, Xi’an, China

**Keywords:** MoP nanowires, Se doping, water-reduction kinetics, electrocatalytic, hydrogen evolution reaction

## Abstract

Owing to its low cost, high conductivity, and chemical stability, Molybdenum phosphide (MoP) has great potential for electrochemically catalyzing the hydrogen evolution reaction (HER). Unfortunately, the development of high-activity MoP still remains a grand challenge in alkali-electrolyzers due to its sluggish water reduction kinetics. Here, we demonstrate a novel strategy for regulating the HER kinetics of the MoP nanowire cathode through partially substituting P atoms with Se dopants. In alkaline solutions, the Se-doped MoP (Se-MoP) nanowire cathode exhibits excellent HER performance with a greatly-decreased overpotential of ∼61 mV at 10 mA cm^−2^ and a Tafel slope of ∼63 mV dec^−1^, outperforming currently reported MoP-based electrocatalysts. Experimental and theoretical investigations reveal that Se doping not only facilitates the water dissociation on MoP, but also optimize the hydrogen adsorption free energy, eventually speeding up the sluggish alkaline HER kinetics. Therefore, this work paves a new path for designing MoP-based electrocatalyst with high HER performance in alkaline electrolyzers.

## Introduction

Because of its large energy density and zero carbon emission, hydrogen (H_2_) has been proposed as the most promising energy carrier for meeting future energy demands (Turner, 2004; Chu and Majumdar, 2012; Chu et al., 2017). Electrochemical water splitting to generate hydrogen is an efficient and sustainable pathway in the future hydrogen economy cycle (Norskov and Christensen, 2006; Seh et al., 2017). Nevertheless, owing to the large energy barrier of water dissociation, exploring highly efficient electrocatalysts is highly desirable for intrinsically improving the reaction kinetics and reducing HER overpotential, especially in alkaline media (Chen et al., 2018; Wang et al., 2020b; Zhu et al., 2020). To date, noble metal-based catalysts (Pt, Ru, etc) are still the benchmark electrocatalysts because of their superior intrinsic HER activity (Lei et al., 2020; Yu et al., 2020). Unfortunately, the expensiveness and rareness of noble metals seriously limit its large-scale utilization in practical electrolyzers (Fang et al., 2020; Xue et al., 2020).

Recently, transition metal phosphides (TMPs), such as MoP show appealing HER performance among various HER electrocatalysts owing to their high conductivity, chemical stability, and similar electronic structure to Pt (Xiao et al., 2014; Yang et al., 2016; Zhang et al., 2018b; Song et al., 2020). Nevertheless, HER activity of MoP is still unsatisfactory compared with those of noble metal-based catalysts, especially in alkaline electrolytes (Jaramillo et al., 2007; Xing et al., 2014; Shi and Zhang, 2016). Therefore, improving the activity and stability of MoP catalysts is urgently demanded in alkaline solutions. Principally, the alkaline HER reaction proceeds via an initial Volmer step (H_2_O + e^−^ → H* + OH^−^) and a sequent Heyrovsky step (H_2_O + e^−^ + H* → H_2_ + OH^−^) or Tafel step (H* + H* → H_2_) (Strmcnik et al., 2016; Wang et al., 2020a). High energy barrier of water dissociation and unamiable hydrogen free energy on MoP catalysts leads to their poor HER performance (Subbaraman et al., 2012; Xu et al., 2017; Chen et al., 2018; Kim et al., 2021). Anion doping has been proven to be a useful method to improve the HER performance of MoP electrocatalysts, including the S-doped MoP nanoporous layer (Liang et al., 2019) and N-doped MoP nanoparticle catalyst (Chen et al., 2020). However, in previous reports, only the water dissociation energy barrier or H* adsorption energy was individually considered to play a critical role in promoting the HER reaction kinetics (Kibsgaard and Jaramillo, 2014; Anjum and Lee, 2017; Liang et al., 2019; Chen et al., 2020). In fact, some electrocatalysts have been designed to reduce both water dissociation energy barrier and H adsorption energy for the enhancement of HER performance, including Ni(OH)_2_/NiMoPO_x_ (Peng et al., 2020), Ni-doped MoS_2_ (Zhang et al., 2016), oxygen-incorporating Co_2_P (Xu et al., 2017), Cr-doped Co_4_N (Yao et al., 2019), etc. Nevertheless, to our best knowledge, it is rarely reported to simultaneously reduce the water-dissociation and hydrogen adsorption energies using anion-doped MoP catalysts toward the HER. In addition, the conventional powdery catalysts should be coated on the current collector using polymeric binder, leading to the inferior conductivity, limited active sites and weak interactions between the catalyst and support (Zhang et al., 2018a; Zhang et al., 2018c; Huang et al., 2019). Accordingly, binder-free MoP could be served as an ideal electrode with high HER performance.

In this work, we develop a phosphorization-selenization process to synthesize Se-doped MoP nanowires consisting of nanoparticles using ultralong Mo nanowires as the Mo source. The Se-MoP nanowires were assembled into a self-supported cathode and performed an excellent HER activity with a low overpotential of ∼61 mV at 10 mA cm^−2^ and a small Tafel slope of ∼63 mV dec^−1^ in 1 M KOH solution, which were superior than pure MoP and previously reported MoP-based electrocatalysts. Experimental investigations and theoretical calculations revealed that Se doping are beneficial for decreasing water dissociation energy and optimizing hydrogen adsorption energy, which ultimately enhanced the sluggish water-reduction kinetics for alkaline hydrogen evolution.

## Experimental Details

### Synthesis of Se-MoP Nanowire Film

A two-temperature zone tube furnace was employed to prepare the Se-MoP nanowire film. The Mo nanowire film (more details in Supplementary Material) was placed into the downstream zone, while NaH_2_PO_2_ or selenium powder was placed in the upstream zone. To synthesize the MoP nanowire film, the temperature of upstream zone was heated up to 300°C and the temperature of the downstream zone was heated up to 800, 900, and 1000°C, respectively. The fabrication processes were carried out for 1 h under a N_2_ atmosphere. When the temperatures were cooled to room temperature, the MoP nanowire film was obtained. The subsequent annealing treatment was performed using selenium powder to replace NaH_2_PO_2_, the temperature for selenium powder was 300°C, and the temperature for the MoP nanowire film was 550°C. To adjust the reaction time, Se-MoP nanowire film with different Se contents was obtained.

### Electrochemical Measurements

Electrocatalytic HER activity of the prepared samples was measured in a CHI760E electrochemical workstation. Three-electrode configuration was equipped with a graphite rod as the counter electrode, Hg/HgO as the reference electrode and MoP and Se-MoP nanowire film as the working electrode. Liner sweep voltammetry (LSV) with a scan rate of 2 mV s^−1^ was conducted in a 1 M KOH aqueous solution. Electrochemical impendence spectroscopy (EIS) was carried out at −0.1 V vs. RHE over a frequency range of 10 kHz–0.01 Hz. Cyclic voltammetry (CV) measurements at different scan rates were performed to determine the electrochemically active specific area (ECSA). RHE potentials were calculated according to the equation: E_RHE_ = E_Hg/HgO_ + 0.099 V + 0.059 V × pH.

### Theoretical Calculations

The Vienna Ab Initio Simulation Package (VASP) code was adopted to carried out all density functional theory (DFT) calculations. (Kresse and Furthmüller, 1996a; Kresse and Furthmüller, 1996b). The Perdew-Burke-Ernzerhof (PBE) functional (Perdew et al., 1996) was used to describe the exchange correlation interaction by the generalized gradient approximation (GGA) method (Blöchl, 1994; Kresse and Joubert, 1999). The kinetic energy cut-off was set to 400 eV and the convergences criteria for the residual forces and energies were set to 0.02 eV/Å and 10^−5^ eV, respectively. A vacuum layer of more than 15 Å was set to avoid the interaction between the periodically repeated slabs. The reaction energetics for hydrogen evolution reaction intermediates were calculated using the computational hydrogen electrode model (Henkelman et al., 2000). For MoP (101) surface, a four-layer of 2*3 supercell was built during the calculation, and for Se doped MoP (101), one P atom on the top surface was doped by Se atom.

## Results and Discussion

**FIGURE 1:**
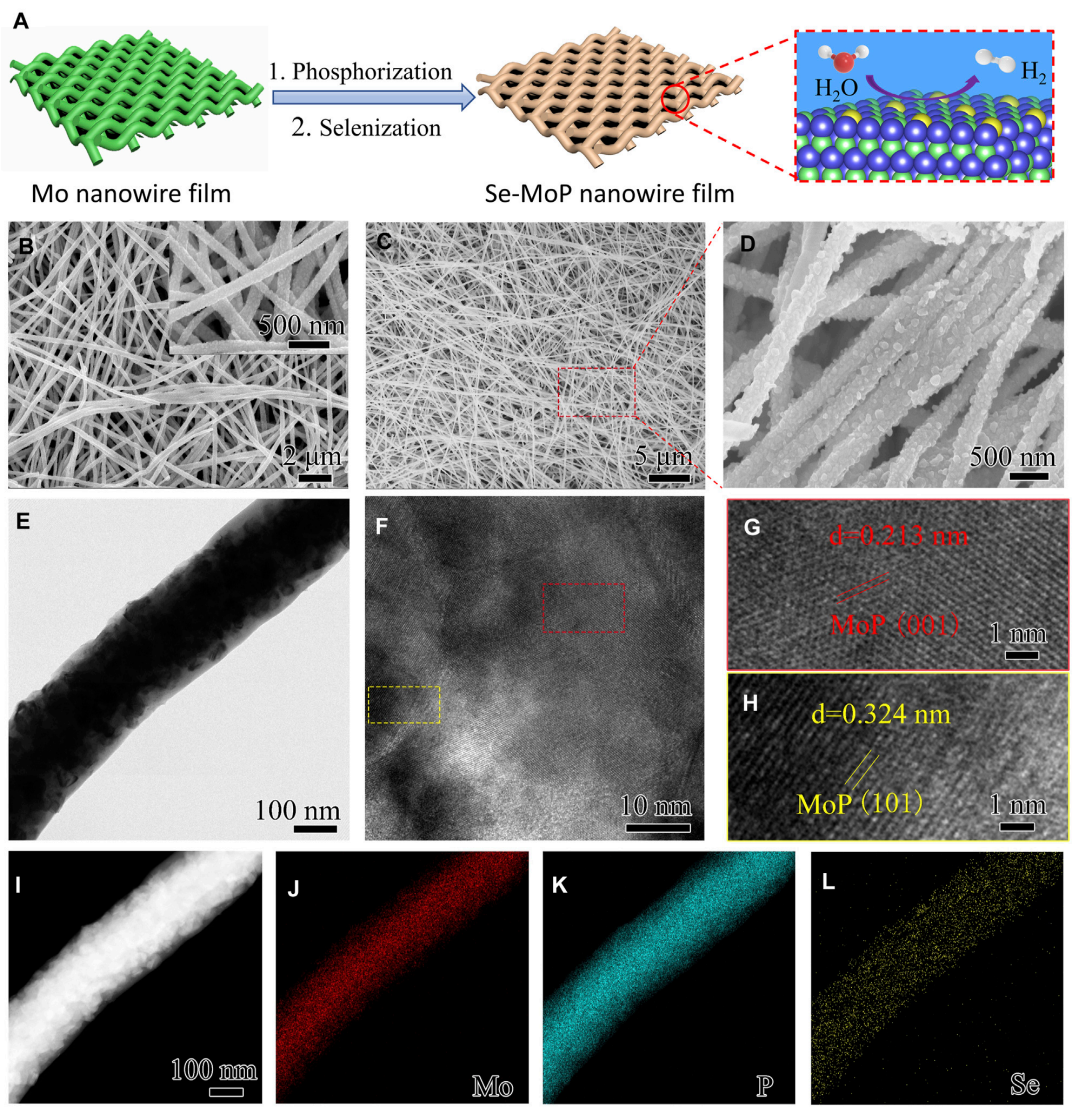
**(A)** Synthetic route of the Se-MoP nanowire film. The blue, green, yellow, red, and white spheres represent Mo, P, Se, O, and H atoms, respectively. **(B)** SEM images of MoP nanowires and **(C**–**D)** Se-MoP nanowires. **(E)** TEM, **(F**–**H)** HRTEM, **(I)** HAADF-STEM images of the Se-MoP nanowires and the corresponding elemental mapping images of **(J)** Mo, **(K)** P, and **(L)** Se elements. The inset in Panel **(B)** represents corresponding high-magnification SEM image of MoP nanowires.

Figure 1A schematically illustrated the fabrication procedure of Se-MoP nanowire film. First, ultralong Mo nanowires (diameter: ∼195 nm, length: >60 μm) were prepared from a directionally solidified NiAl-9Mo eutectic alloy by selective etching and then were assembled into a self-supported film through vacuum filtration (Supplementary Figure S1) (Liu et al., 2020). Second, MoP nanowires with sizes of 20–60 nm nanoparticles were obtained after phosphorization treatment at 900°C for 1 h using NaH_2_PO_2_ as a phosphorus source (Figure 1B, Supplementary Figures S2, S3). Eventually, after a half-hour annealing treatment of precursor MoP nanowire film at 550°C in Se vapor environments, the Se-MoP nanowire film was achieved. Se-doped MoP samples with different Se contents were prepared by changing selenization time.

The microstructures of MoP and Se-MoP nanowire electrocatalysts were evaluated by scanning electron microscopy (SEM) and transmission electron microscopy (TEM). As displayed in Figures 1C,D and Supplementary Figure S4, both MoP and Se-MoP nanowires were composed of nanoparticles with the size from 30 to 50 nm. TEM image of Se-MoP in Figure 1E further confirmed the nanowire structure with a larger diameter of ∼253 nm than the Mo nanowires, which was due to the growth of MoP. Subsequently, high-resolution TEM images show two interplanar spacings of 0.213 and 0.324 nm (Figures 1G,H), which are in accord with the (101) and (001) planes of MoP (Du et al., 2017; Xiao et al., 2020), respectively. The lattice space of Se-MoP nanowires was larger than the pure MoP (Supplementary Figure S5), indicating that the doping of Se could cause lattice expansion of MoP. Supplementary Figure S6 displays the diffraction patterns of the (001) and (101) facets of MoP. In addition, the elemental mapping of Se-MoP nanowires in Figure 1I demonstrates the uniform distribution of Mo, P and Se element. The Se content in Se-MoP nanowires (Se-MoP-30 min) was estimated to be ∼3.42 at% according to the EDX spectrum in Supplementary Figure S7. The morphologies of the Se-MoP nanowires under different selenization time were investigated. As disclosed in Supplementary Figure S8, all Se-MoP samples showed similar nanowire morphology. The atomic contents of Se in Se-MoP nanowires were found to be ∼1.27 and ∼5.94 at% (Supplementary Table S1) after 15-min (Se-MoP-15 min) and 60-min (Se-MoP-60 min) selenization.

**FIGURE 2:**
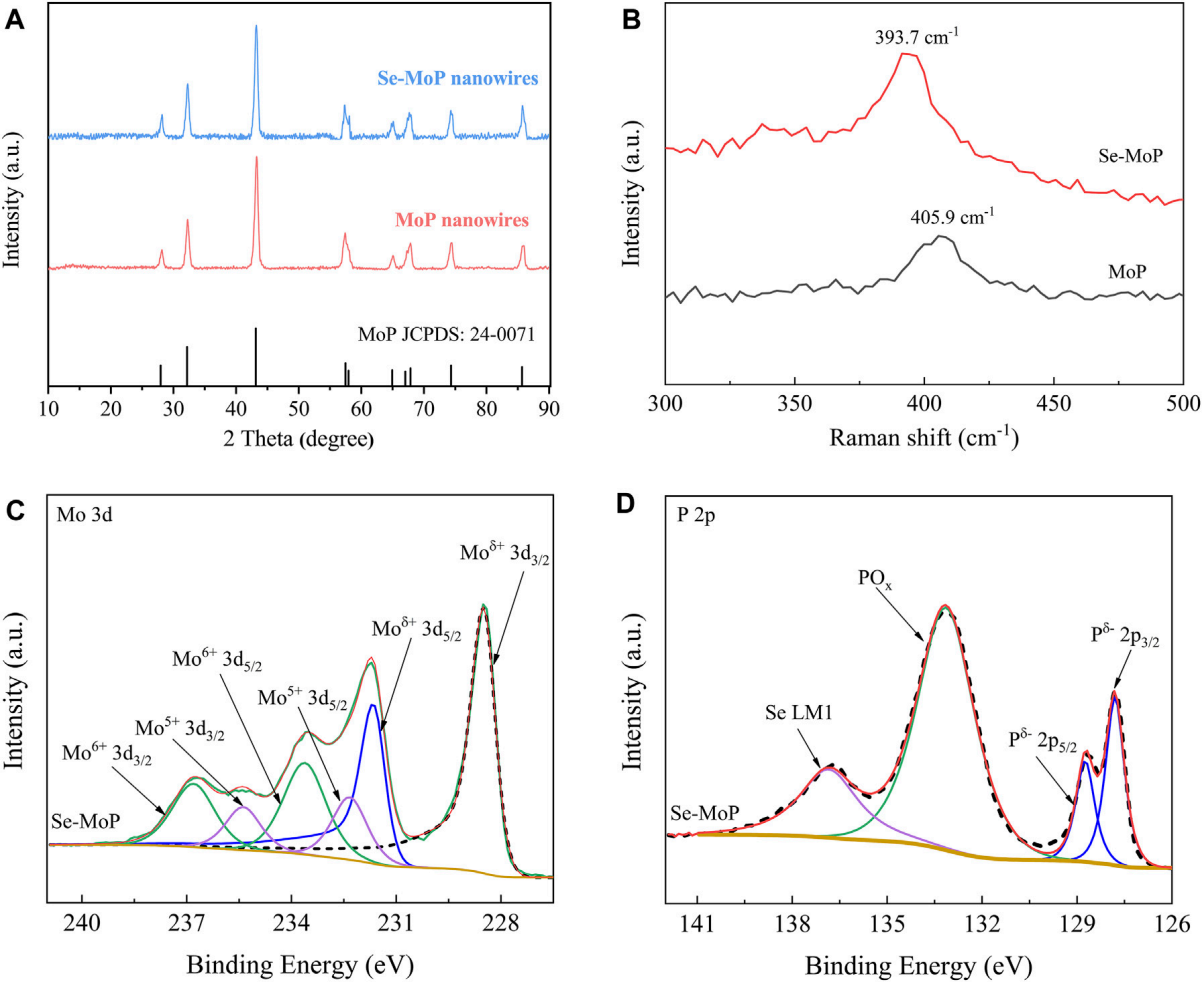
**(A)** XRD patterns, **(B)** Raman spectra, **(C)** high-resolution XPS spectra of Mo 3 days, and **(D)** P 2p of Se-MoP nanowires.

The crystal information of the catalysts was evaluated by X-ray diffraction (XRD). As shown in Figure 2A and Supplementary Figure S9A, for both MoP and Se-MoP nanowires, the diffraction peaks located at 27.9°, 32.1°, 43.2°, 57.3°, 57.7°, 64.8°, 67.6°, 74.3°, and 85.7° originated from the (001), (100), (101), (110), (002), (111), (102), (201), and (112) planes of the hexagonal MoP phase (JCPDS 24-0071) (Liu et al., 2018; Zhang et al., 2018b; Zhang et al., 2018c), respectively. Noticeably, no peaks of Se-related phases could be detected in the Se-MoP nanowires. Along with increased Se contents, the peak of the (101) facet shifted slightly to smaller 2-*θ* angles compared with pure MoP (Supplementary Figure S9B), implying that the Se doping caused lattice expansion of the MoP. This result agreed well with the HRTEM results. Then, the MoP and Se-MoP phases were further investigated through Raman spectroscopy. (Figure 2B). Clearly, the distinct peak of the stretching mode in Se-MoP at 393.7 cm^−1^ shifted to a low wavenumber compared to pure MoP (405.9 cm^−1^) (Yin et al., 2016).

For investigating the valence state of un-doped and doped MoP electrocatalysts, X-ray photoelectron spectroscopy (XPS) was carried out. As shown in Supplementary Figure S10, the Mo, P and Se elements exist in Se-MoP nanowires. Figure 2C and Supplementary Figure S11 present the high-resolution spectra of the Mo 3d peaks of the Se-MoP and MoP nanowires. The peaks of Se-MoP nanowires located at 228.5 and 231.7 eV, which were attributed to ${\text{Mo}}^{\text{δ}+}$ (3d_5/2_) ( 0<δ<4 ) and (3d_3/2_) of MoP (Chen et al., 2020; Xiao et al., 2020), respectively. The other two doublet peaks located at relatively higher binding energies of 232.3/235.4 and 233.6/236.9 eV were corresponding to Mo^5+^ 3d_5/2_/Mo^5+^ 3d_3/2_ and Mo^6+^ 3d_5/2_/Mo^6+^ 3d_3/2_, due to surface oxidation of Mo (Chen et al., 2017). The high-solution P 2p spectrum of Se-MoP in Figure 2D has two peaks at 129.7 and 130.6 eV, which originates from ${\text{p}}^{\text{δ}-}$ (3p_3/2_) and ${\text{p}}^{\text{δ}-}$ (3p_5/2_) of Mo-P bonds in MoP (Ye et al., 2016), respectively. The peak positioned at 134.7 eV could be assigned to PO_x_ species caused by slightly oxidized P (Liu et al., 2021). The Se-MoP nanowires displayed a peak at 138.7 eV, which could be derived from Se LM1. In the XPS Se 3d spectra (Supplementary Figure S12) of Se-MoP nanowires, peaks at 54.5 and 55.3 eV were associated with Se (3d_3/2_) and Se (3d_5/2_) of Mo-Se bonds (Wang et al., 2014). The above SEM, TEM, XRD, Raman, and XPS results together demonstrate that the Se atoms were successfully doped into MoP nanowires with a uniform distribution by partially substituting P atoms.

**FIGURE 3:**
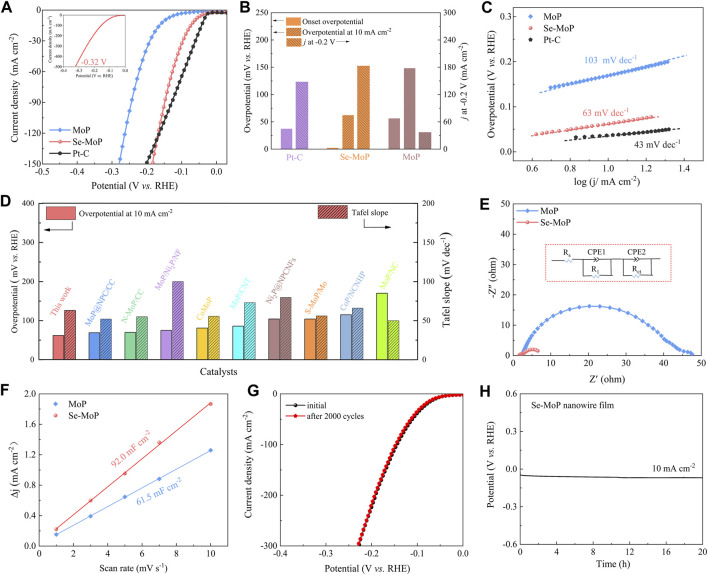
Electrochemical investigations of Se-MoP nanowires, MoP nanowires and commercial Pt/C electrocatalysts in 1 M KOH solution. **(A)** LSV curves. **(B)** Comparison of various catalysts in onset potential, overpotential at 10 mA cm^−2^ and current densities at −0.2 V vs*.* RHE. **(C)** The corresponding Tafel plots of various catalysts. **(D)** Comparison of the overpotential and Tafel slope among the Se-MoP nanowires and reported transition metal phosphate-based electrocatalysts under alkaline conditions. **(E)** Nyquist plots (−0.1 V vs*.* RHE) of MoP and Se-MoP nanowires in 1 M KOH. **(F)** Double-layer capacitances. **(G)** LSV curves of Se-MoP nanowires before and after 2,000 cyclic voltammetry cycles. **(H)** Chronopotentiometric measurement of Se-MoP nanowires at 10 mA cm^−2^.

The HER activity of MoP nanowire film and Se MoP nanowire film was assessed in a 1 M KOH solution. The commercial Pt/C loaded on a nickel foam was used as the reference catalyst. All LSV curves were iR compensated. As depicted in Figures 3A,B, the Se-MoP nanowire cathode exhibited an onset HER potential of ∼2 mV and an overpotential of ∼61 mV at 10 mA cm^−2^, which were much lower than pure MoP nanowires (∼148 mV) and those for previously reported MoP-based HER electrocatalysts, such as N-doped MoP/CC (∼70 mV) (Chen et al., 2020), CoMoP@N-doped C (∼81 mV) (Ma et al., 2017), S-doped MoP nanoporous layer (∼104 mV) (Liang et al., 2019), carbon-coated Ni-doped MoP (∼162 mV) (Xiao et al., 2020), and MoP nanoparticles/NC (∼170 mV) (Huang et al., 2019) (Supplementary Table S2). Apparently, the Se-MoP nanowire cathode achieved a very large current density of 184 mA cm^−2^ at −0.2 V, which was approximately 4.95 times higher than that for the MoP nanowire. More importantly, the Se-MoP nanowires only required an overpotential of 320 mV for delivering an industrial current density of 500 mA cm^−2^ (inset in Figure 3A).

As displayed in Figure 3C, the Tafel plots of various electrocatalysts were investigated. Obviously, the Se-MoP nanowires presented a small Tafel slope of 63 mV dec^−1^, much lower than 103 mV dec^−1^ for MoP nanowires. As generally accepted, HER mechanism was ascribed to the following two pathways in alkaline electrolyte (Strmcnik et al., 2016): the first step is an electrochemical reduction (Volmer step: H_2_O + e^–^ + *→ H* + OH^−^) with a Tafel slop of 118 mV dec^−1^. And the second step is either the ion and atom reaction (Heyrovsky step: H* + H_2_O + e^−^ → H_2_ + OH^−^ + *) or the atom combination reaction (Tafel step: H* + H* → H_2_ + 2*), corresponding to a slopes of 39 or 29 mV dec^−1^, respectively. Therefore, the low slope (63 mV dec^−1^) on Se-MoP catalysts suggested a possible Volmer-Heyrovsky mechanism, and the H* desorption to gaseous H_2_ is the rate-determining step (Man et al., 2011). Meanwhile, the exchange current density (*j*_*0*_) of Se-MoP catalysts was determined to be 1.193 mA cm^−2^, which was higher than 0.251 mA cm^−2^ for MoP (Supplementary Figure S13), suggesting a superior electrocatalytic activity.

To probe the reason of the excellent electrocatalytic performance, the electrochemical impedance spectroscopy (EIS) of MoP and Se-MoP nanowires film was first investigated at −0.1 V. Based on the Nyquist plots (Figure 3E), the charge transfer resistance (*R*_*ct*_) of the Se-MoP nanowires was fitted to be ∼6.03 Ω, which was lower than ∼46.14 Ω for MoP nanowires, indicating that the doping of Se facilitated the electron transport during the HER. Furthermore, combining with CV scans at different scan rates (Supplementary Figure S14), electrochemical double-layer capacitances (C_dl_) were obtained to determine the electrochemical active surface area (ECSA), which was proportional to the ECSA (Zhu et al., 2020). As disclosed in Figure 3F, the C_dl_ of the Se-MoP nanowires was 92.0 mF cm^−2^, which was higher than that of the MoP nanowires (61.5 mF cm^−2^). This result indicates that Se doping generates more exposed active sites. Then, the LSV curves was further normalized versus their ESCA (Supplementary Figure S15). Clearly, at 0.15 mA cm^−2^, the HER overpotential of the Se-MoP nanowires was 268 mV, which was much lower than 318 mV for the MoP nanowires. Thus, the active sites in Se-MoP nanowires are intrinsically high-activity. The turnover frequency (TOF) is an important parameter for evaluating the inherent activity of the electrocatalysts. As illustrated in Supplementary Figure S16, the Se-MoP nanowires acquired a considerably higher TOF value of ∼0.229 s^−1^ at an overpotential of 100 mV than 0.023 s^−1^ for MoP nanowires.

To investigate the influence of Se contents on the electrocatalytic performance of the MoP nanowires, we further assessed the electrochemical performance of different Se-MoP catalysts with different Se contents. As shown in Supplementary Figure S17A, the Se-MoP nanowires with 3.42 at% Se afforded an overpotentials of 61 mV at a current density of 10 mA cm^−2^ compared to 114 mV for Se-MoP with 1.27 at% Se and 105 mV for Se-MoP with 5.94 at% Se, respectively. Even under large current densities like 100 and 200 mA cm^−2^ (Supplementary Figure S17B), the Se-MoP nanowires with 3.42 at% Se still exhibited the smallest overpotential of ∼144 and 191 mV, respectively. Therefore, the superior HER activity of the Se-MoP nanowires is ascribed to the optimized Se doping.

For assessing the electrocatalytic stability of Se-MoP catalyst, we first conducted 2000 CV cycles between 0.025 V and −0.375 V at a scan rate of 50 mV s^−1^. Apparently, the HER overpotential showed a negligible decline at the same current density compared to the initial state (Figure 3G). Subsequently, the long-term stability tests for 20 h were conducted in 1 M KOH (Figure 3L). The Se-MoP nanowires retained a steady HER overpotential over a period of 20 h. After the long-term stability measurement, the Se-MoP nanowires was further investigated using the XRD, SEM, TEM, and the corresponding EDX mapping characterizations (Supplementary Figures S18–S20). Clearly, the structural morphology and chemical composition of Se-MoP nanowires remain well, suggesting outstanding stability during the HER process.

**FIGURE 4:**
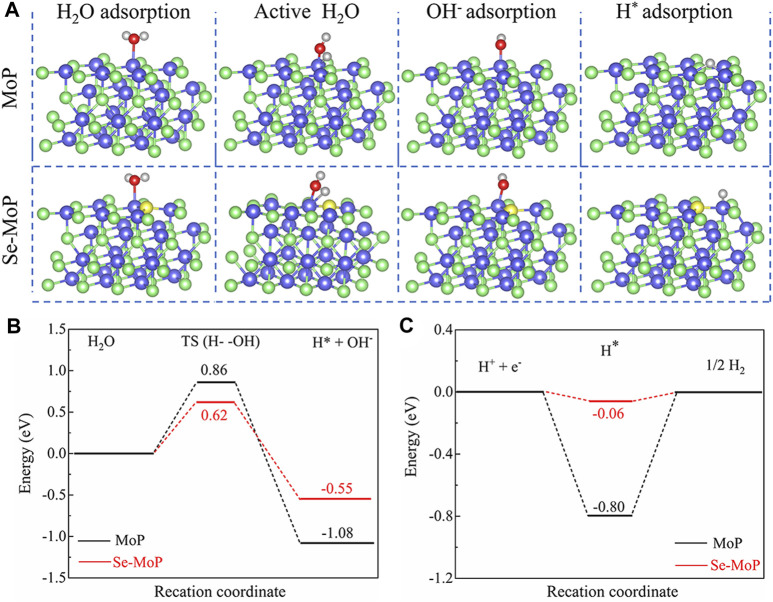
DFT calculations of pure MoP and Se doped MoP. **(A)** Calculation models of the surface, H_2_O adsorption, OH^−^ adsorption and H* adsorption of MoP and Se-MoP. The blue, green, yellow, red, and gray spheres represent Mo, P, Se, O, and H atoms, respectively. **(B)** The calculated activation energy barriers of H_2_O molecules and **(C)** adsorption energies of H* on MoP (black) and Se-MoP surfaces (red).

To gain deep insights into the enhanced mechanism of HER for Se doped MoP nanowires, density functional theory (DFT) calculations of MoP and Se-MoP were conducted (Figure 4A). We first calculated the initial water dissociation energy barriers (Volmer step). The Se-MoP showed a dramatically decreased water dissociation energy barrier [Δ*G* (H_2_O)] of 0.62 eV, which was much lower than the value of 0.86 eV for MoP (Figure 4B). Additionally, we also calculated the hydrogen adsorption free energy (ΔG_H_), whose value close to 0 eV indicated a high HER activity (Greeley et al., 2006). As shown in Figure 4C, the ΔG_H_ (−0.06 eV) of Se-MoP was much closer to 0 eV relative to the ΔG_H_ of MoP (−0.80 eV). Therefore, these theoretical results demonstrate that promoting Volmer and Heyrovsky processes on Se-MoP leads to a rapid water-reduction kinetics toward excellent HER performance in alkaline electrolyte.

## Conclusion

In summary, we synthesized a Se-doped MoP nanowire cathode through a phosphorization-selenization treatment using a Mo nanowire film as the precursor. Compared to those for preciously reported MoP-based electrocatalysts, the Se-MoP nanowire cathode shows greatly improved HER activity with a lower overpotential of ∼61 mV at 10 mA cm^−2^ in alkaline solution. As revealed by the experimental results and DFT calculations, the incorporation of Se boosts water dissociation and optimizes hydrogen free energy adsorption on MoP, thus accelerating alkaline HER kinetics. The study affords a new approach for designing high-performance transition metal phosphides electrocatalysts for HER.

## Data Availability

The original contributions presented in the study are included in the article/Supplementary Material, further inquiries can be directed to the corresponding authors.
