# Supplementary material for: Regulating Water Reduction Kinetics on MoP Electrocatalysts Through Se Doping for Accelerated Alkaline Hydrogen Production
Source: Front Chem. 2021 Oct 1;9:737495. doi: 10.3389/fchem.2021.737495 (PMC8517518; doi:10.3389/fchem.2021.737495)
Supplement: Supplementary file 1 [file DataSheet1.docx]

Supplementary Material

**Regulating water reduction kinetics on MoP electrocatalysts through Se doping for accelerated alkaline hydrogen production**

**Zhenpeng Liu ^1, 2^, Jun Bu ^2^, Wenxiu Ma ^2^, Bin Yang ^1^, Lei Zhang ^2,^ *, Hong Zhong ^1,^ *, Shuangming Li ^1^, Jian Zhang ^2^**

^1^State Key Laboratory of Solidiﬁcation Processing, Northwestern Polytechnical University, Xi' an, 710129 P. R. China

^2^Key Laboratory of Special Functional and Smart Polymer Materials of Ministry of Industry and Information Technology and Department of Advanced Chemical Engineering, School of Chemistry and Chemical Engineering, Northwestern Polytechnical University, Xi’an 710129, P. R. China

*** Correspondence:**Corresponding Author: Hong Zhong, Lei Zhang
[leizhang@nwpu.edu.cn](mailto:leizhang@nwpu.edu.cn)

[zhonghong123@nwpu.edu.cn](mailto:zhonghong123@nwpu.edu.cn)

**Experimental Section**

***Materials***

Ni plate (99.99%), Al rod (99.99%) and Mo plate (99.99%) were purchased from Beijing Guanjinli Ltd. (China). Sodium hypophosphite (NaH_2_PO_2_, 98%) and selenium powder (Se, 99%) were purchased from Aladdin (China). Commercial 20 wt% Pt/C and Nafion solution (5 wt%) were purchased from Shanghai Hesen (China). Ni foam was bought from KunShan Kuangxun Ltd. (China). The ultrapure water (>18.25 MΩ cm) was used for the experiments.

**Preparation of Mo nanowire film**

Ultralong Mo nanowires were fabricated through selective dissolution of NiAl phase in a directionally solidified NiAl-9Mo (45.5 at% Ni-45.5 at% Al-9 at% Mo) eutectic alloy according to our previous report method. NiAl-9Mo ingots were prepared using the vacuum induction melting furnace with pure elements of Ni, Al and Mo. A rod with a diameter of 10 mm and a length of 100 mm was cut from the NiAl-9Mo ingot and placed into an alumina crucible of 10/11 mm in diameter (inside/outside diameter) and 120 mm in length for directional solidification in an improved Bridgman-type furnace. The specimen was heated to 1900 ± 10 K for 1 h and thermal stabilized for 20 min. Then, it was directionally solidified with a growth rate of 180 cm h^-1^ at a thermal gradient of approximately 200 K cm^-1^. Selective dissolution of directionally solidified NiAl-9Mo rod was performed in a mix solution of HCl (36 wt%), H_2_O_2_ (30 wt%) and H_2_O (10 : 10 : 80; v/v/v) for 24 h at room temperature to ensure that the NiAl phase was completely etched. After washing with deionized water and drying at 60 ℃, large-scale Mo nanowires (~1.46 mg) were achieved. Eventually, 100 mg Mo nanowires were dispersed into 50 ml deionized (DI) water and treated for 30 min under ultrasound. Above suspension was then assembled onto a nylon membrane (pore size: 0.44 μm; diameter: 4 cm) using vacuum filtration. After drying for 30 min at 60 ℃, a self-supported Mo nanowire film was easily peeled off.

**Materials characterization**

The XRD patterns were recorded by using Cu Kα radiation by X-ray diffractometry (XRD, PANalytical B.V., Netherlands). The surface phase composition of the samples was also studied by employing the Raman (Alpha300R). Scanning electron microscopy (FESEM, FEI-Verios G4) was applied to analyze the morphologies of the prepared electrocatalysts. Transmission electron microscopy (TEM) and high-resolution TEM (HRTEM) images were acquired on FEI Talos F200X at an acceleration voltage of 200 kV. The X-ray photoelectron spectroscopy (XPS, Kratos-Axis Supra) was carried out to decide the chemical composition and valence state of element of the eletrocatalysts.


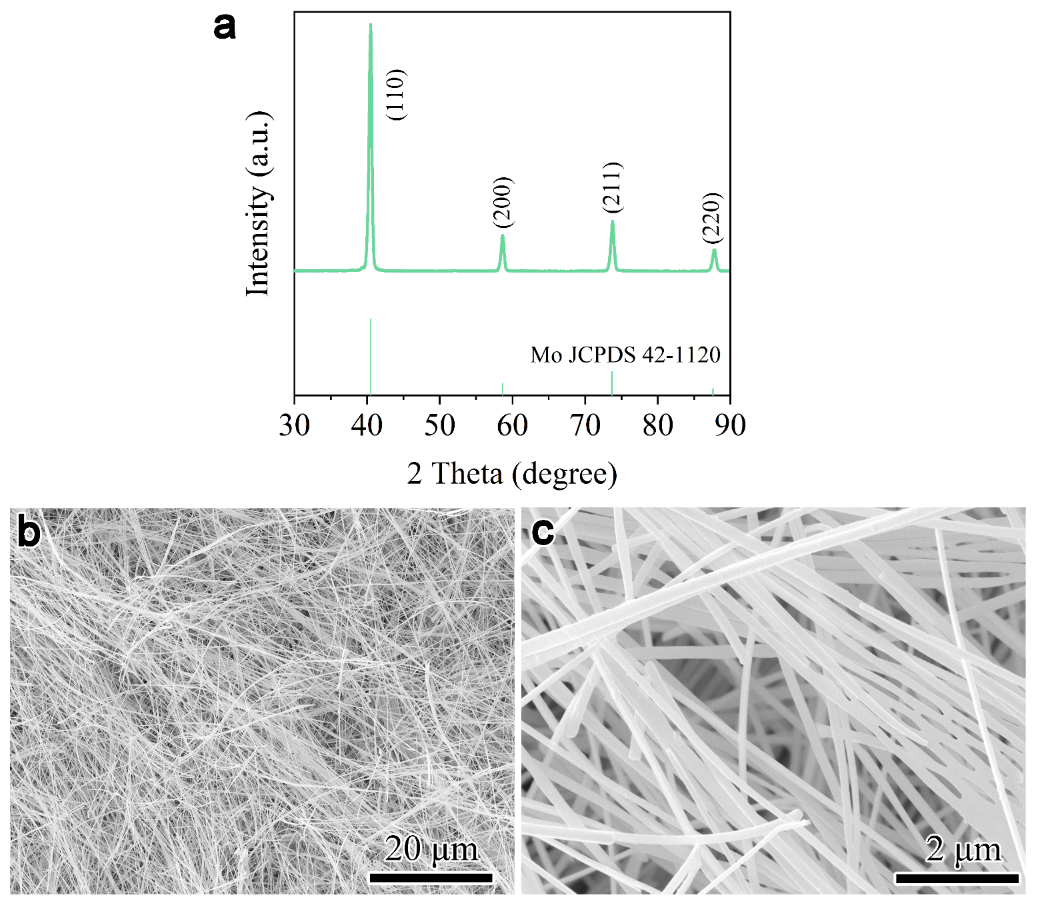


**Figure S1.** (a) XRD patterns of Mo nanowire film. (b-c) SEM images of Mo nanowires.





**Figure S2.** XRD patterns of MoP nanowire film obtained at different temperatures.


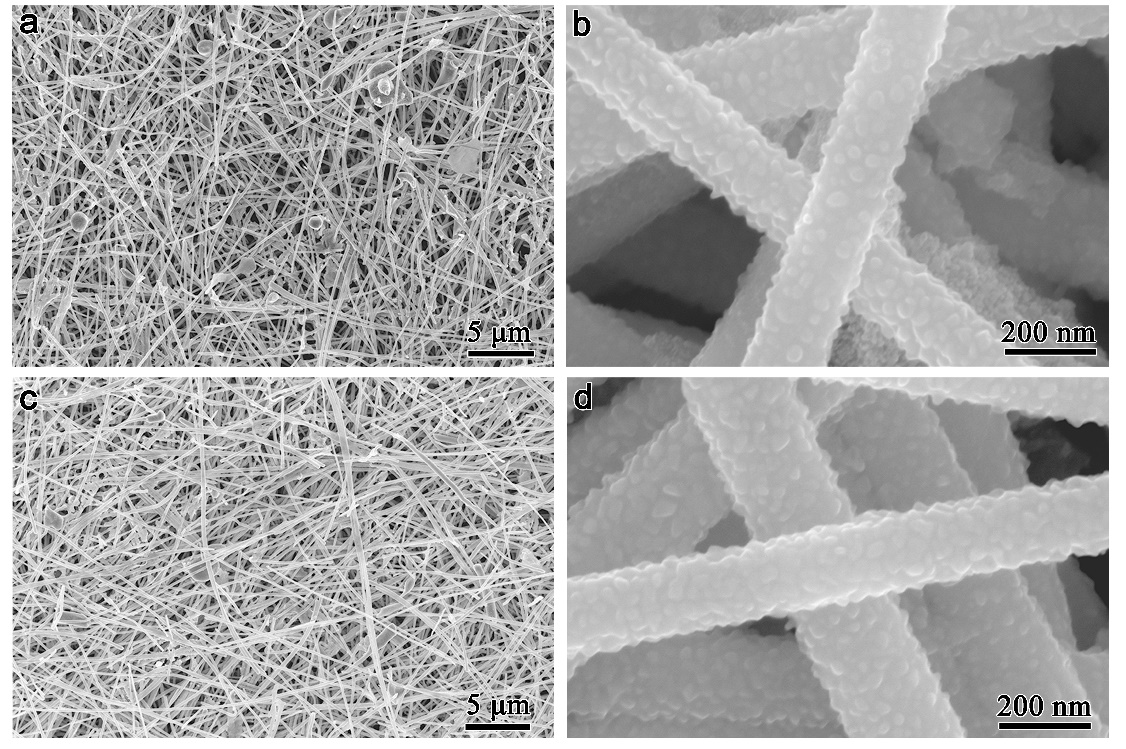


**Figure S3.** SEM images of MoP at 800 ℃ (a-b) and 1000 ℃ (c-d).


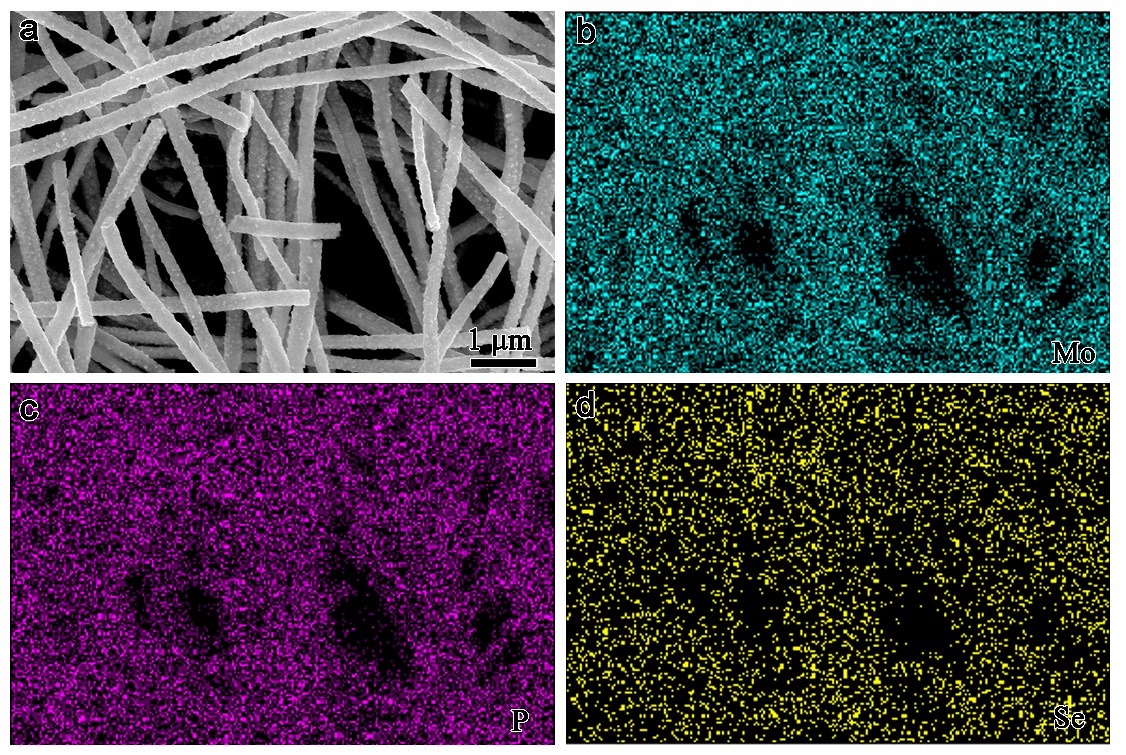


**Figure S4.** (a) SEM images and corresponding elemental images of the Se-MoP nanowires, (b) Mo, (c) P and (d) Se elements.


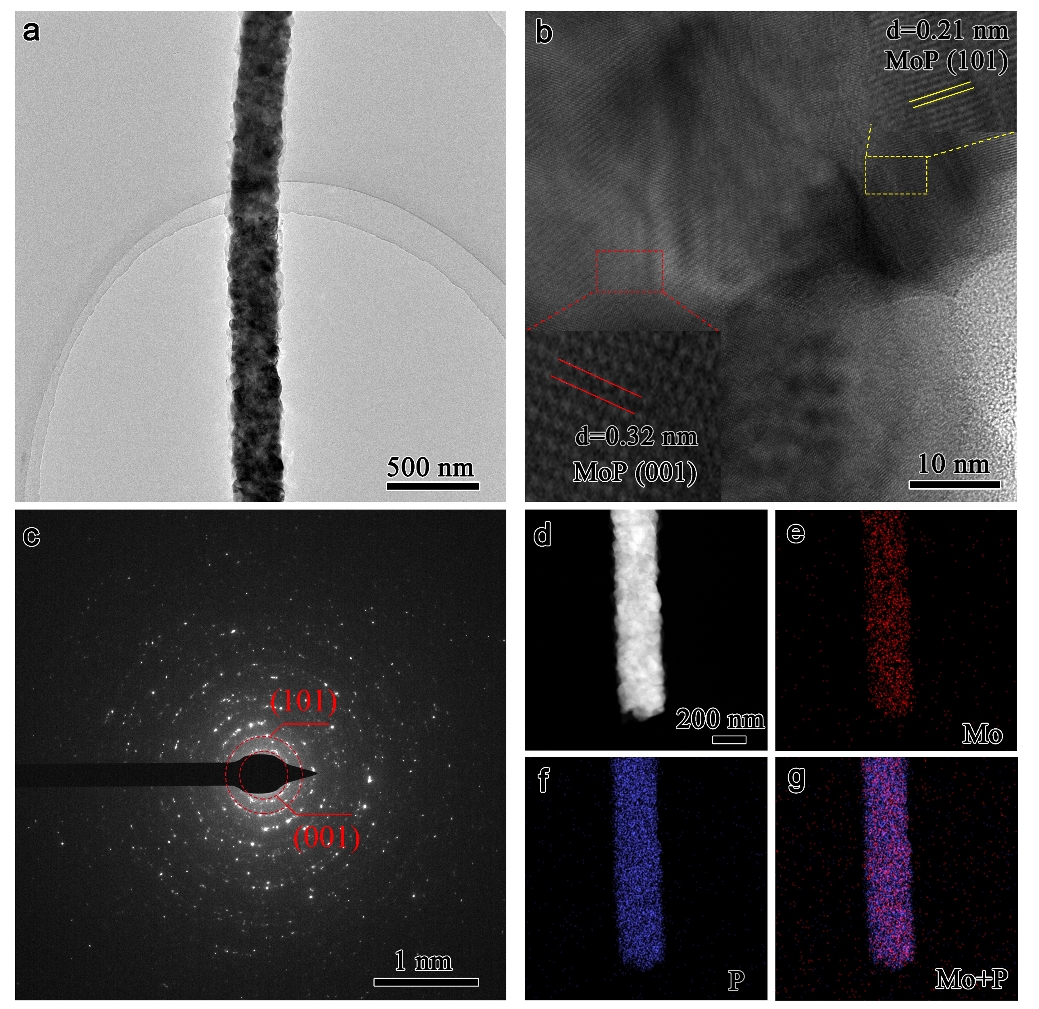


**Figure S5.** (a) TEM images, (b) HRTEM images and (c) SAED patterns of MoP nanowires. (d) HAADF-STEM image of MoP nanowires. The corresponding elemental mapping images of (e) Mo, (f) P and (g) Mo+P elements in MoP nanowires.


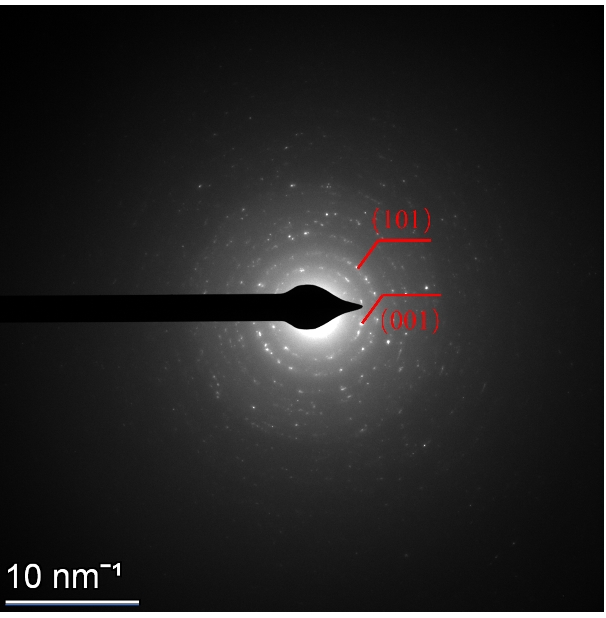


**Figure S6.** SAED pattern of Se-MoP nanowires.


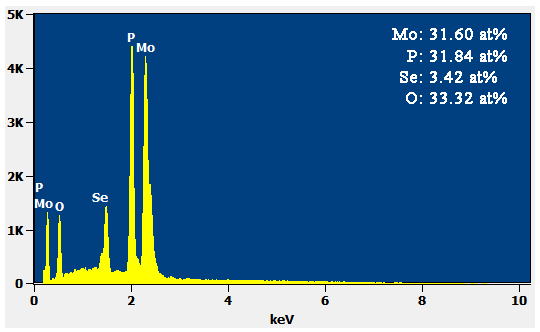


**Figure S7.** EDX spectrum of Se-MoP nanowires.


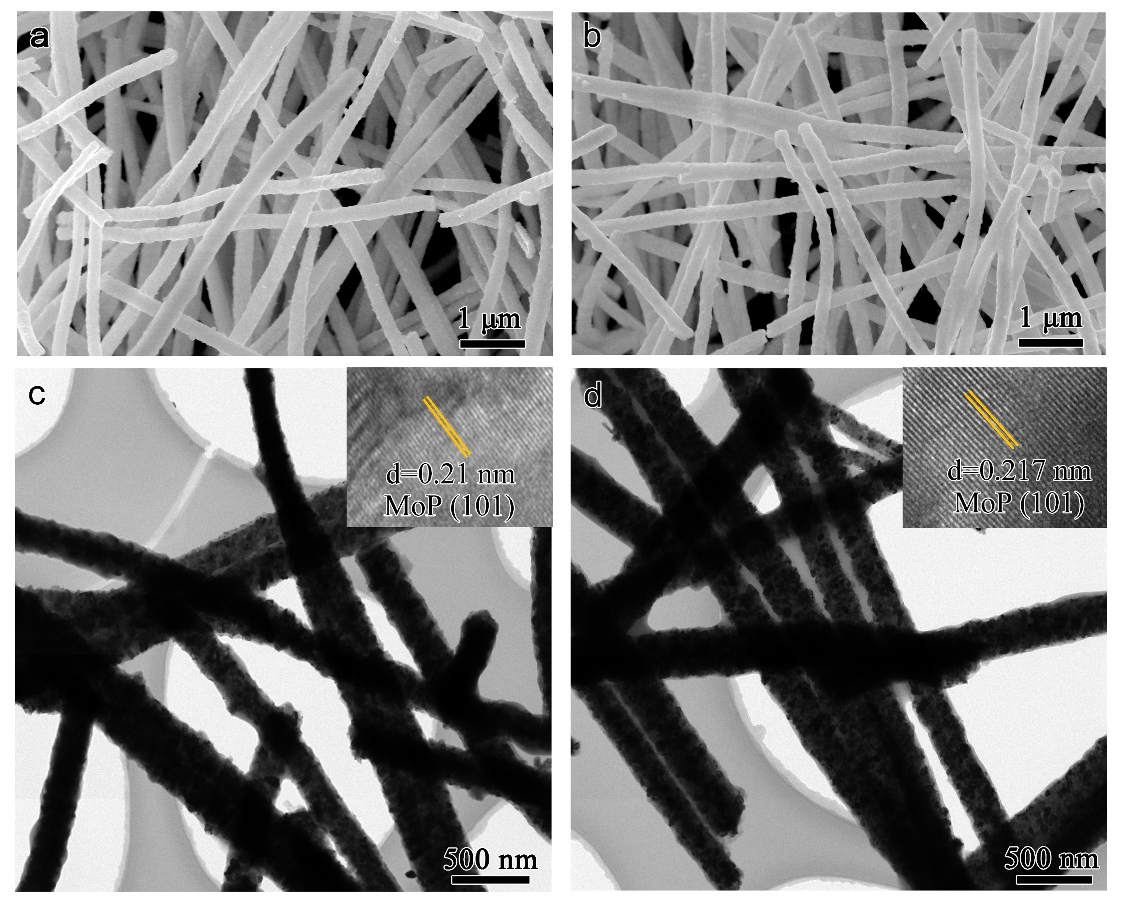


**Figure S8.** SEM images of Se-MoP-15 min (a) and Se-MoP-60 min nanowires (b). TEM images of Se-MoP-15 min (c) and Se-MoP-60 min nanowires (d). The insets in Figure S8c and d are the related HRTEM images of Se-MoP-15 min and Se-MoP-60 min nanowires.

**Table S1.** The element contents in different MoP nanowires.

| Catalyst | Mo (at%) | P (at%) | Se (at%) | O (at%) |
| --- | --- | --- | --- | --- |
| Se-MoP-15 min | 30.82 | 31.99 | 1.27 | 35.92 |
| Se-MoP-30 min | 31.60 | 31.84 | 3.42 | 33.32 |
| Se-MoP-60 min | 28.38 | 28.94 | 5.94 | 36.74 |


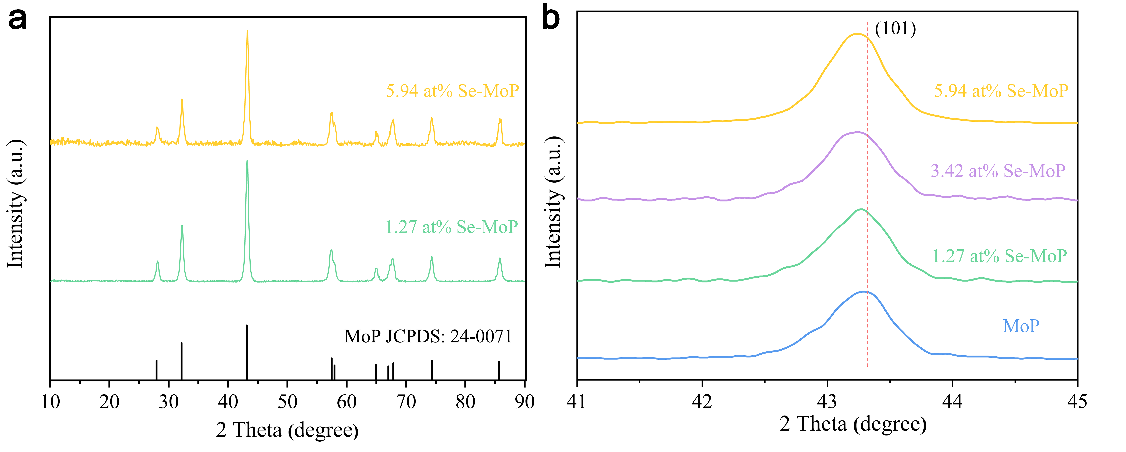


**Figure S9.** (a) XRD patterns of Se-MoP-15 min and Se-MoP-60 min nanowires, (b) The enlarged region of (101) plane for MoP and Se-MoP nanowires.





**Figure S10.** XPS survey spectrum of Se-MoP nanowires.

**
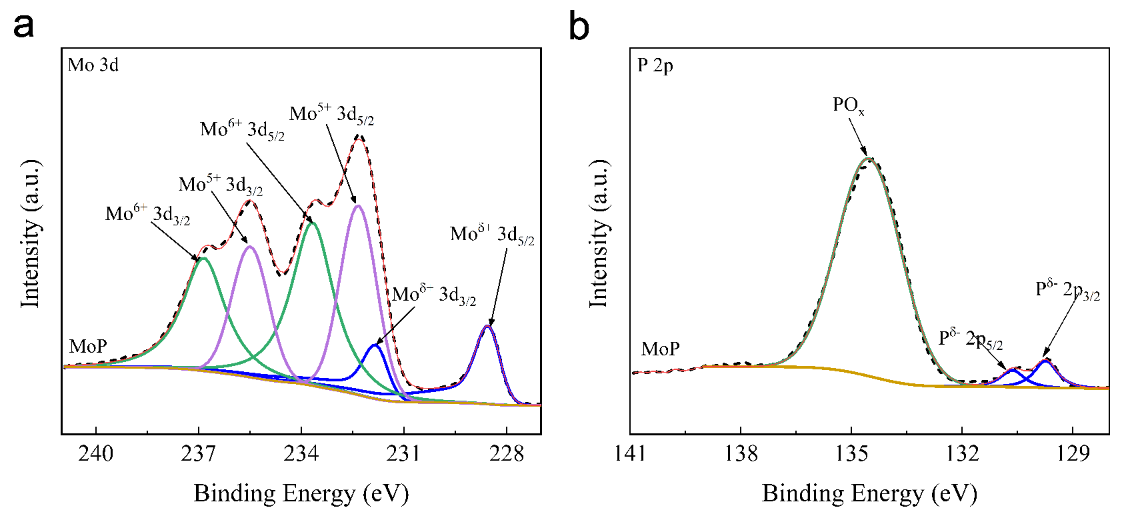
**

**Figure S11.** High-resolution XPS spectra of Mo 3d and (d) P 2p of MoP nanowires.

**

**

**Figure S12.** High-resolution XPS spectra of Se 3d of Se-MoP nanowires.

**Table S2.** Comparison of the HER performance of Se-MoP nanowires with previously reported HER electrocatalysts.

| Catalysts | Overpotential  (mV at 10 mA cm^-2^) | Tafel slope  (mV dec^-1^) | Electrolyte | References |
| --- | --- | --- | --- | --- |
| Se-MoP nanowires | 61 | 63 | 1 M KOH | This work |
| MoP nanoparticles/NC | 170 | 50 | 1 M KOH | Appl. Catal B. **2019**, 245, 656 |
| N-MoP/CC | 70 | 55 | 1 M KOH | Appl. Catal B. **2020**, 268, 118411 |
| S-doped MoP nanoporous layer | 104 | 56 | 1 M KOH | ACS Catal. **2019**, 9, 651 |
| Carbon  -coated Ni-doped MoP | 162 | 102.6 | 1 M KOH | Nano Energy. **2020**, 70, 104445 |
| MoP/CNT hybrid | 86 | 73 | 1 M KOH | Adv. Funct. Mater. **2018**, 28, 1706523 |
| N, P dual-doped carbon encapsulated MoP/CC | 69 | 52 | 1 M KOH | Adv. Funct. Mater. **2018**, 28, 1801527 |
| CoMoP@N-doped C | 81 | 55.53 | 1 M KOH | Energy Environ. Sci. **2017**, 10, 788 |
| CoP@N-doped carbon tube hollow polyhedron | 115 | 66 | 1 M KOH | J. Am. Chem. Soc. **2018**, 140, 2610 |
| Ni_2_P@N-doped porous carbon | 104.2 | 79.7 | 1 M KOH | Angew. Chem. Int. Ed. **2018**, 130, 1981 |
| MoP/Ni_2_P on 3D Ni foam | 75 | 100 | 1 M KOH | J. Mater. Chem. A **2017**, 5, 15940 |
| Carbon-dots-loaded MoP | 70 | 77.49 | 1 M KOH | Nano Energy. **2020**, 72, 104730 |
| FeP_2_ nanowires/Fe foil | 189 | 67 | 1 M KOH | Chem. Commun. **2016**, 52, 2819 |
| WP nanowires | 150 | 102 | 1 M KOH | ACS Appl. Mater. Inter. **2014**, 6, 21874 |





**Figure S13.** The exchange current densities of Se-MoP nanowires, MoP nanowires and commercial Pt/C on Ni foam.


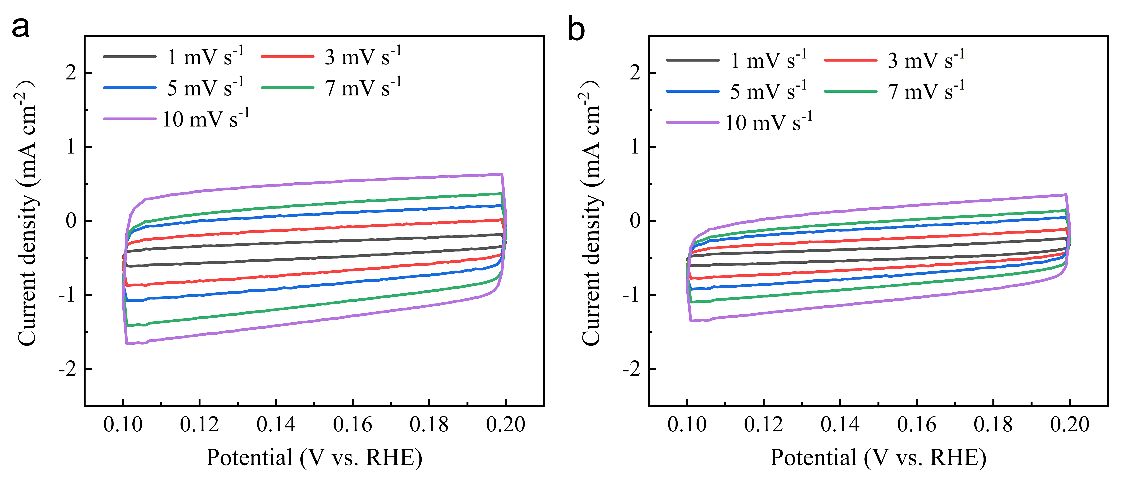


**Figure S14.** Cyclic voltammograms (CV) were recorded in a potential range (0.1~0.2 V vs. RHE) without faradic processes for (a) Se-MoP nanowires and (b) MoP nanowires.





**Figure S15.** LSV curves normalized to the electrochemical active surface area (ECSA) for MoP and Se-MoP nanowires.





**Figure S16.** TOFs of MoP and Se-MoP nanowires at different overpotentials.


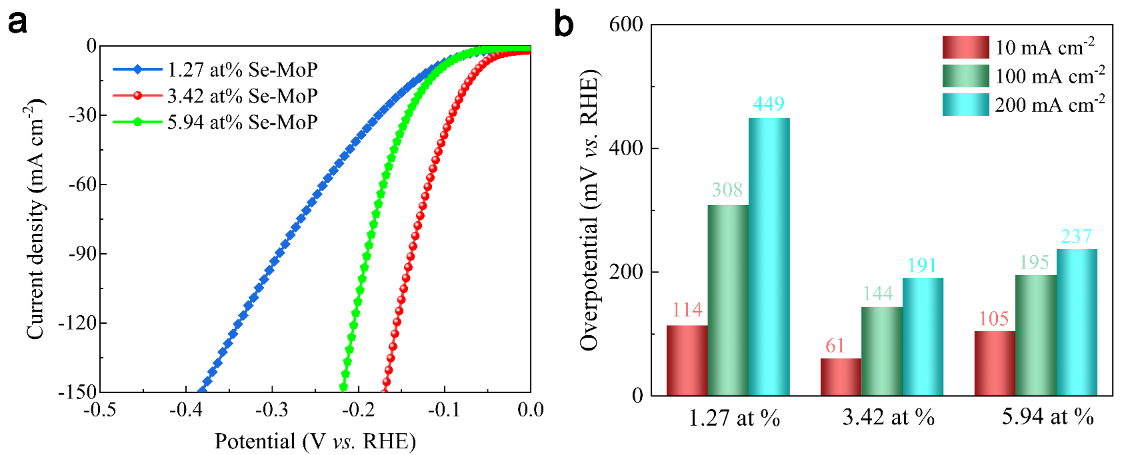


**Figure S17.** HER performance of MoP nanowire film with different Se contents. (a) LSV curves and (b) overpotentials at different current densities.





**Figure S18.** The XRD pattern of Se-MoP nanowires after the long-term stability test.


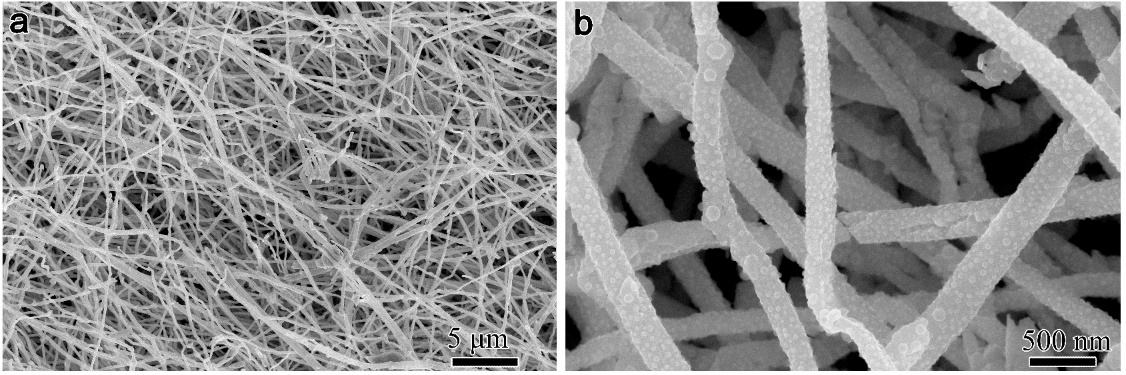


**Figure S19.** The SEM images of Se-MoP nanowires after the long-term stability tests.


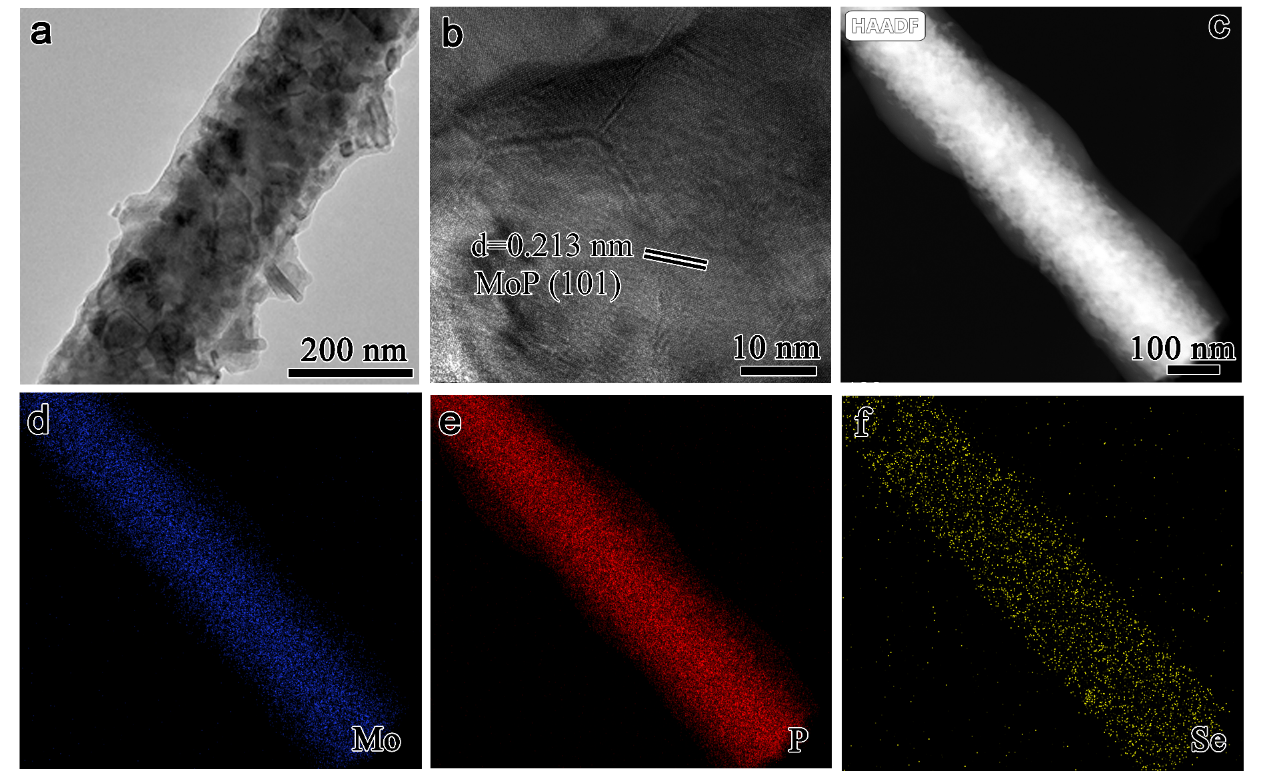


**Figure S20.** (a) TEM, (b) HRTEM, and (c) HAADF-STEM images of Se-MoP nanowires after the long-term stability tests. The corresponding elemental mapping images of (d) Mo, (e) Ni and (f) Se elements in Se-MoP nanowires.
